# Portal protein diversity and phage ecology

**DOI:** 10.1111/j.1462-2920.2008.01702.x

**Published:** 2008-10

**Authors:** Matthew B Sullivan, Maureen L Coleman, Vanessa Quinlivan, Jessica E Rosenkrantz, Alicia S DeFrancesco, G Tan, Ross Fu, Jessica A Lee, John B Waterbury, Joseph P Bielawski, Sallie W Chisholm

**Affiliations:** 1 Departments of Civil and Environmental EngineeringCambridge, MA 02139, USA; 2 Departments of Biology, Massachusetts Institute of TechnologyCambridge, MA 02139, USA; 3 Department of Biology, Woods Hole Oceanographic InstitutionWoods Hole, MA 02543, USA; 4 Department of Biology, Dalhousie UniversityNova Scotia, Canada

## Abstract

Oceanic phages are critical components of the global ecosystem, where they play a role in microbial mortality and evolution. Our understanding of phage diversity is greatly limited by the lack of useful genetic diversity measures. Previous studies, focusing on myophages that infect the marine cyanobacterium *Synechococcus*, have used the coliphage T4 portal-protein-encoding homologue, gene 20 (*g20*), as a diversity marker. These studies revealed 10 sequence clusters, 9 oceanic and 1 freshwater, where only 3 contained cultured representatives. We sequenced g20 from 38 marine myophages isolated using a diversity of *Synechococcus* and *Prochlorococcus* hosts to see if any would fall into the clusters that lacked cultured representatives. On the contrary, all fell into the three clusters that already contained sequences from cultured phages. Further, there was no obvious relationship between host of isolation, or host range, and g20 sequence similarity. We next expanded our analyses to all available g20 sequences (769 sequences), which include PCR amplicons from wild uncultured phages, non-PCR amplified sequences identified in the Global Ocean Survey (GOS) metagenomic database, as well as sequences from cultured phages, to evaluate the relationship between g20 sequence clusters and habitat features from which the phage sequences were isolated. Even in this meta-data set, very few sequences fell into the sequence clusters without cultured representatives, suggesting that the latter are very rare, or sequencing artefacts. In contrast, sequences most similar to the culture-containing clusters, the freshwater cluster and two novel clusters, were more highly represented, with one particular culture-containing cluster representing the dominant g20 genotype in the unamplified GOS sequence data. Finally, while some g20 sequences were non-randomly distributed with respect to habitat, there were always numerous exceptions to general patterns, indicating that phage portal proteins are not good predictors of a phage's host or the habitat in which a particular phage may thrive.

Virus-like particles occur in high abundance (to 10^8^ ml^−1^) in the oceans (Bergh, 1989; Bratbak *et al*., 1990; Proctor and Fuhrman, 1990). One of the most well-studied phage–host systems in this habitat is the phages that infect the marine cyanobacteria *Prochlorococcus* and *Synechococcus*, which are globally important marine primary producers (Waterbury *et al*., 1986; Partensky *et al*., 1999). These ‘cyanophages’ are abundant (Waterbury and Valois, 1993; Suttle and Chan, 1994; Suttle, 2000; Lu *et al*., 2001; Frederickson *et al*., 2003; Marston and Sallee, 2003; Sullivan *et al*., 2003), contribute to host mortality (Waterbury and Valois, 1993; Suttle and Chan, 1994; Suttle, 2000) and are thought to play a role in maintaining the extensive microdiversity of their hosts (Waterbury and Valois, 1993; Suttle and Chan, 1994; Marston and Sallee, 2003; Sullivan *et al*., 2003) likely through killing the winner (*sensu*Thingstad, 2000) and through the movement of genes throughout the host population (Lindell *et al*., 2004; Coleman *et al*., 2006; Sullivan *et al*., 2006).

Studying the diversity of phages has proven difficult because no universal gene, analogous to the 16S rRNA gene used for microbes, exists throughout all phage families (Paul *et al*., 2002). Thus family-specific genes have been proposed for use as taxonomic tools in phage ecology (Rohwer and Edwards, 2002). One such marker, a homologue to the coliphage T4 portal protein gene 20 (g20), has been developed to study the diversity of *Myoviridae –* one of the most common phage types observed in metagenomics surveys (Breitbart *et al*., 2002; 2004a; DeLong *et al*., 2006) and among *Synechococcus* cyanophage isolates (Suttle and Chan, 1993; Waterbury and Valois, 1993; Wilson *et al*., 1993; Sullivan *et al*., 2003). The g20 homologue is ubiquitous among T4-like myoviruses (see T4-like phages genome website http://phage.bioc.tulane.edu/) with hosts ranging from proteobacteria to cyanobacteria (Fuller *et al*., 1998; Hambly *et al*., 2001; Mann *et al*., 2005; Sullivan *et al*., 2005). The evolution of g20 is likely constrained because its protein product initiates capsid assembly (at least in T4), a process which involves geometric precision (Coombs and Eiserling, 1977; van Driel and Couture, 1978; Hsiao and Black, 1978) through the formation of a proximal vertex (van Driel and Couture, 1978) used for DNA packaging (Hsiao and Black, 1978) and binding the capsid to the tail junction (Coombs and Eiserling, 1977).

The availability of cultured cyanomyophage (Waterbury and Valois, 1993; Wilson *et al*., 1993; Suttle and Chan, 1993; Marston and Sallee, 2003; Sullivan *et al*., 2003) has allowed the design of cyanomyophage-specific g20 sequence PCR primers that have been used to study this component of viral populations in the wild. Early studies using non-degenerate PCR primers and DNA ‘fingerprinting’ techniques (e.g. denaturing gradient gel electrophoresis and terminal-restriction fragment length polymorphism banding patterns) revealed variability in g20 diversity across gradients in space and time from a variety of different environments (Wilson *et al*., 1999; 2000; Frederickson *et al*., 2003; Dorigo *et al*., 2004; Wang and Chen, 2004; Mühling *et al.*, 2005; Sandaa and Larsen, 2006). These studies concluded that g20 diversity was as great within a sample as between oceans (Wilson *et al*., 1999), that phage g20 diversity increased as *Synechococcus* abundance increased (Wilson *et al*., 1999; 2000; Frederickson *et al*., 2003; Wang and Chen, 2004; Sandaa and Larsen, 2006), that some g20 types were ubiquitous in the habitats examined (Wilson *et al*., 1999; 2000; Frederickson *et al*., 2003; Dorigo *et al*., 2004), as well as a temporal study by Muhling andcolleagues (2005) that correlated ‘cyanophage’ diversity (inferred from g20 sequence types) with *Synechococcus* diversity (inferred from *rpoC1* sequence types).

Subsequent cloning and sequencing of g20 PCR amplicons from both cultured isolates and wild populations have allowed phylogenetic analyses of cyanomyophage diversity. Although initial studies (Zhong *et al*., 2002) suggested some correlation between ocean habitat and g20 phylogeny (e.g. phylogenetic cluster II represents ‘open ocean’ g20 sequences), further sampling revealed that this was not the case, as seven g20 sequences from coastal *Synechococcus* myophages isolated from Rhode Island waters clustered with the putative ‘open ocean’ sequences (Marston and Sallee, 2003). As more g20 sequence data have accumulated from diverse environments (Zhong *et al*., 2002; Marston and Sallee, 2003; Dorigo *et al*., 2004; Short and Suttle, 2005; Sandaa and Larsen, 2006; Wilhelm *et al*., 2006), it has become clear that marine g20 sequences form nine phylogenetic clusters (first described by Zhong *et al*., 2002), and g20 sequences originating from freshwater environments form a separate, tenth cluster (Dorigo *et al*., 2004; Short and Suttle, 2005; Wilhelm *et al*., 2006). Three of the nine marine clusters (clusters I–III in Zhong *et al*., 2002) contain cultured representatives (hereafter called ‘culture-containing clusters’), whereas the remaining six marine clusters (clusters A-F) and the ‘freshwater’ cluster do not (hereafter called ‘environmental-sequence-only clusters’). The cultured representatives were isolated using only *Synechococcus* hosts (7 strains = WH7803, WH7805, WH8007, WH8012, WH8018, WH8101, WH8113), which undoubtedly limits the diversity represented considering the larger diversity of *Synechococcus* strains (Rocap *et al*., 2002; Fuller *et al*., 2003; Ahlgren and Rocap, 2006) and that the sister genus *Prochlorococcus* is also abundant in open ocean waters. This raises the question: could these seven environmental-sequence-only clusters represent novel cyanomyophages that infect this broader diversity of *Synechococcus* host strains, *Prochlorococcus* or other cyanobacteria?

To address this question, we isolated phages on a broad diversity of *Prochlorococcus* and *Synechococcus* hosts (Table 1), sequenced their g20 homologues and analysed their diversity in the context of published PCR-generated sequences from natural populations. We then combined the g20 sequences from these new cultured isolates with all environmental g20 sequences available [including all PCR-generated environmental sequences, as well as primer-independent sequences available in the Global Ocean Survey (GOS) metagenomic data set], to examine the broad diversity of g20 observed in the wild. This allowed us to ask: do any of the new environmental sequences cluster with the previously observed environmental-sequence-only clusters? Furthermore, are g20 sequence clustering patterns ecologically meaningful? Do they reflect the habitat – and by inference the microbial community – of the site from which they were isolated?

**Table 1:** Efficacy of three different primer sets at amplifying the g20 gene from cultured cyanophage.

						g20 primer set			
Phage strain	Original host strain isolated on	Site of Isolation	Depth (m)	Date isolated	Family^a^	CPS4GC/5	CPS1/8	CPS1.1/8.1	Refs^b^
*Prochlorococcus* cyanophage									
P-SSP1	MIT 9215	BATS/31°48′N, 64°16′W	100	6 June 2000	P	–	–	–	1
P-RSP1	MIT 9215	Red Sea/29°28′N, 34°53′E	0	15 July 2000	P	–	–	–	1
P-RSP2	MIT 9302	Red Sea/29°28′N, 34°53′E	0	15 July 2000	P	–	–	–	1
P-SSP2	MIT 9312	BATS/31°48′N, 64°16′W	120	29 September 1999	P	–	–	–	1
P-SSP3	MIT 9312	BATS/31°48′N, 64°16′W	100	29 September 1999	P	–	–	–	1
P-SSP4	MIT 9312	BATS/31°48′N, 64°16′W	70	26 September 1999	P	–	–	–	1
P-SSP5	MIT 9515	BATS/31°48′N, 64°16′W	120	29 September 1999	P	–	–	–	1
P-SSP6	MIT 9515	BATS/31°48′N, 64°16′W	100	26 September 1999	P	–	–	–	1
P-SSP7	MED4	BATS/31°48′N, 64°16′W	100	26 September 1999	P	–	–	–	1
P-GSP1	MED4	Gulf Stream/38°21′N, 66°49′W	40	6 October 1999	P	–	–	–	1
P-SSP8	NATL2A	BATS/31°48′N, 64°16′W	100	26 September 1999	P	–	–	–	1
P-RSP3	NATL2A	Red Sea/29°28′N, 34°55′E	50	13 September 2000	P	–	–	–	1
P-SP1	SS120	Slope/38°10′N, 73°09′W	83	17 September 2001	P	–	–	–	1
**P-SSM8**	**MIT 9211**	**W Sargasso Sea/34°24′N, 72°03′W**	**30**	**22 September 2001**	**M**	**+**	**+**	**+**	**2**
**P-SSM1**	**MIT 9303**	**BATS/31°48′N, 64°16′W**	**100**	**6 June 2000**	**M**	**+**	**–**	**+**	**1**
**P-RSM1**	**MIT 9303**	**Red Sea/29°28′N, 34°53′E**	**0**	**15 July 2000**	**M**	**+**	**–**	**+**	**1**
**P-RSM4**	**MIT 9303**	**Red Sea/29°28′N, 34°55′E**	**130**	**13 September 2000**	**M**	**+**	**+**	**+**	**2**
**P-ShM1**	**MIT 9313**	**Shelf/39°60′N, 71°48′W**	**40**	**16 September 2001**	**M**	**+**	**–**	**+**	**1**
**P-ShM2**	**MIT 9313**	**Shelf/39°60′N, 71°48′W**	**0**	**16 September 2001**	**M**	**–**	**–**	**+**	**1**
**P-SSM2**	**NATL1A**	**BATS/31°48′N, 64°16′W**	**100**	**6 June 2000**	**M**	**+**	**+**	**+**	**1**
**P-RSM5**	**NATL1A**	**Red Sea/29°28′N, 34°55′E**	**130**	**13 September 2000**	**M**	**+**	**+**	**+**	**2**
**P-SSM7**	**NATL1A**	**BATS/31°48′N, 64°16′W**	**120**	**29 September 1999**	**M**	**–**	**–**	**+**	**2**
**P-SSM3**	**NATL2A**	**BATS/31°48′N, 64°16′W**	**100**	**6 June 2000**	**M**	**–**	**–**	**+**	**1**
**P-SSM4**	**NATL2A**	**BATS/31°48′N, 64°16′W**	**10**	**6 June 2000**	**M**	**–**	**–**	**+**	**1**
**P-SSM5**	**NATL2A**	**BATS/31°48′N, 64°16′W**	**15**	**26 September 1999**	**M**	**+**	**–**	**+**	**1**
**P-SSM6**	**NATL2A**	**BATS/31°48′N, 64°16′W**	**40**	**29 September 1999**	**M**	**–**	**–**	**+**	**1**
**P-RSM2**	**NATL2A**	**Red Sea/29°28′N, 34°55′E**	**50**	**13 September 2000**	**M**	**+**	**–**	**+**	**1**
**P-RSM3**	**NATL2A**	**Red Sea/29°28′N, 34°55′E**	**50**	**13 September 2000**	**M**	**–**	**–**	**+**	**1**
**P-SSM9**	**NATL2A**	**W Sargasso Sea/34°24′N, 72°03′W**	**0**	**22 September 2001**	**M?**	**+**	**–**	**+**	**2**
**P-SSM10**	**NATL2A**	**W Sargasso Sea/34°24′N, 72°03′W**	**0**	**22 September 2001**	**M?**	**+**	**–**	**+**	**2**
**P-SSM11**	**NATL2A**	**W Sargasso Sea/34°24′N, 72°03′W**	**0**	**22 September 2001**	**M?**	**+**	**–**	**+**	**2**
**P-SSM12**	**NATL2A**	**W Sargasso Sea/34°24′N, 72°03′W**	**95**	**22 September 2001**	**M?**	**+**	**–**	**+**	**2**
*Synechococcus* cyanophage									
Syn5	WH 8109	Sargasso Sea/36°58′N, 73°42′W	0	December 1990	P	–	–	–	1
Syn12	WH 8017	Gulf Stream/34°06′N, 61°01′W	0	July 1990	P	–	–	–	1
**S-SM1**	**WH 6501**	**Slope/38°10′N, 73°09′W**	**0**	**17 September 2001**	**M**	**–**	**–**	**+**	**1**
**S-ShM1**	**WH 6501**	**Shelf/39°60′N, 71°48′W**	**0**	**16 September 2001**	**M**	**+**	**+**	**+**	**1**
**S-SSM1**	**WH 6501**	**W Sargasso Sea/34°24′N, 72°03′W**	**70**	**22 September 2001**	**M**	**+**	**+**	**+**	**1**
**Syn 2**	**WH 8012**	**Sargasso Sea/34°06′N, 61°01′W**	**0**	**July 1990**	**M**	**–**	**+**	**+**	**3**
**Syn 9**	**WH 8012**	**Woods Hole/41°31′N, 71°40′W**	**0**	**October 1990**	**M**	**+**	**+**	**+**	**3**
**Syn 10**	**WH 8017**	**Gulf Stream/36°58′N, 73°42′W**	**0**	**December 1990**	**M**	**+**	**+**	**+**	**3**
**Syn 26**	**WH 8017**	**NE Providence Channel/25°53′N, 77°34′W**	**0**	**January 1992**	**M**	**+**	**+**	**+**	**3**
**S-SM2**	**WH 8017**	**Slope/38°10′N, 73°09′W**	**15**	**17 September 2001**	**M**	**+**	**–**	**+**	**2**
**Syn30**	**WH 8018**	**NE Providence Channel/25°53′N, 77°34′W**	**0**	**January 1992**	**M**	**+**	**–**	**+**	**3**
**S-SSM3**	**WH 8018**	**W Sargasso Sea/34°24′N, 72°03′W**	**0**	**22 September 2001**	**M**	**+**	**+**	**+**	**2**
**S-SSM4**	**WH 8018**	**W Sargasso Sea/34°24′N, 72°03′W**	**110**	**22 September 2001**	**M**	**+**	**+**	**+**	**2**
S-RIM3	WH 8018	Mt. Hope Bay, RI/41°39′N, 71°15′W	0	September 1999	M?	+	–	+	4
**Syn 33**	**WH 7803**	**Gulf Stream/25°51′N, 79°26′W**	**0**	**January 1995**	**M**	**+**	**+**	**+**	**3**
S-PM2	WH 7803	English Channel/50°18′N, 4°12′W	0	23 September 1992	M	+	+	+	5
S-WHM1	WH 7803	Woods Hole/41°31′N, 71°40′W	0	11 August 1992	M	+	+	+	5
S-RIM9	WH 7803	Mt. Hope Bay, RI/41°39′N, 71°15′W	0	May 2000	M?	+	–	+	4
S-RIM17	WH 7803	Mt. Hope Bay, RI/41°39′N, 71°15′W	0	July 2001	M?	+	–	+	4
S-RIM24	WH 7803	Mt. Hope Bay, RI/41°39′N, 71°15′W	0	December 2001	M?	+	–	+	4
S-RIM30	WH 7803	Mt. Hope Bay, RI/41°39′N, 71°15′W	0	June 2002	M?	+	–	+	4
**Syn 1**	**WH 8101**	**Woods Hole/41°31′N, 71°40′W**	**0**	**August 1990**	**M**	**+**	**–**	**+**	**3**
**S-ShM2**	**WH 8102**	**Shelf/39°60′N, 71°48′W**	**0**	**16 September 2001**	**M**	**+**	**+**	**+**	**1**
**S-SSM2**	**WH 8102**	**W Sargasso Sea/34°24′N, 72°03′W**	**0**	**22 September 2001**	**M**	**+**	**+**	**+**	**1**
**S-SSM5**	**WH 8102**	**W Sargasso Sea/34°24′N, 72°03′W**	**95**	**22 September 2001**	**M**	**+**	**+**	**+**	**2**
**Syn 19**	**WH 8109**	**Sargasso Sea/34°06′N, 61°01′W**	**0**	**July 1990**	**M**	**–**	**–**	**+**	**3**
**S-SSM6**	**WH 8109**	**W Sargasso Sea/34°24′N, 72°03′W**	**70**	**22 September 2001**	**M**	**+**	**+**	**+**	**2**
**S-SSM7**	**WH 8109**	**W Sargasso Sea/34°24′N, 72°03′W**	**95**	**22 September 2001**	**M**	**+**	**+**	**+**	**2**
Other phages									
IH6-φ1	IH6	Inner Harbor, Baltimore, MD	0	17 November 2000	M	–	–	–	6
IH6-φ7	IH6	Inner Harbor, Baltimore, MD	0	17 November 2000	P	–	–	–	6
IH11-φ2	*Alteromonas*	Inner Harbor, Baltimore, MD	0	17 November 2000	M	–	–	–	6
IH11-φ5	*Alteromonas*	Inner Harbor, Baltimore, MD	0	17 November 2000	P	–	–	–	6
CB8-φ2	CB8	Chesapeake Bay, MD	0	17 November 2000	M	–	–	–	6
CB8-φ6	CB8	Chesapeake Bay, MD	0	17 November 2000	M	–	–	–	6
CB-φ8	*Vibrio alginolyticus*	Chesapeake Bay, MD	0	17 November 2000	M	–	–	–	6
HER320	H7	Helgoland, North Sea	0	1976−1978	M	–	–	–	7
HER321	H100	Helgoland, North Sea	0	1976−1978	P	–	–	–	7
HER322	H100	Helgoland, North Sea	0	1976−1978	M	–	–	–	7
HER327	11–68	Helgoland, North Sea	0	1976−1978	S	–	–	–	7
HER328	H105	Helgoland, North Sea	0	1976−1978	S	–	–	–	7

a M, P and S represent the virus families *Myoviridae*, *Podoviridae* and *Siphoviridae* respectively, as determined by morphology. ‘M?’ indicates that the assignment is based solely on amplification and sequencing of a g20 PCR product and has not been confirmed with electron microscopy.

b Reference where cultured isolate was originally described: 1, Sullivan and colleagues (2003); 2, this study; 3, Waterbury and Valois (1993); 4, Marston and Salee (2003); 5, Wilson and colleagues (1993); 6, Zhong and colleagues (2002); 7, Wichels and colleagues (1998).

‘+’ indicates positive PCR amplification; ‘−’ indicates that there was no PCR product of the expected size. The new g20 sequences contributed in this study are shown in bold letters. CPS1.1/8.1 is the new primer set designed for this study, while CPS4GC/5 and CPS1/8 were published previously.

## Results and discussion

### Analysis of g20 diversity captured by several g20 primer sets

As our understanding of marine myoviruses has grown over the years, multiple primer sets have been developed and used to specifically amplify cyanomyophage g20 sequences from field samples (Fuller *et al*., 1998; Wilson *et al*., 1999; 2000; Zhong *et al*., 2002; Frederickson *et al*., 2003; Marston and Sallee, 2003; Dorigo *et al*., 2004; Wang and Chen, 2004; Sandaa and Larsen, 2006; Wilhelm *et al*., 2006). Each of these primer sets was designed based on a limited number of sequences from cultured isolates. Thus we wondered how well these primer sets would capture the diversity of g20 sequences in our relatively extensive *Prochlorococcus* and *Synechococcus* cyanophage collection (Table 1).

We found that the CPS4GC/5 primer set (Wilson *et al*., 1999) amplified g20 sequences from 80% of the cyanomyophages screened (bold entries in Table 1). This primer set, however, amplifies only a small region of this gene (∼165 bp), thus its utility for subsequent phylogenetic analyses is limited. In contrast, the CPS1/8 primer set (Zhong *et al*., 2002), which captures a larger segment of the gene (∼594 bp), amplified the g20 sequence of only 56% of the cyanomyophages screened (Table 1). Using genome sequence data from two *Prochlorococcus* cyanomyophages (Sullivan *et al*., 2005) that became available after these primer sets were designed, we modified the CPS1/8 primer set with the hope of amplifying g20 from all of our isolates for use in subsequent phylogenetic analyses. Indeed, the redesigned set (CPS1.1/8.1) captured g20 homologues from all cyanomyophage isolates screened (Table 1). Despite their degeneracy, the redesigned primer set remained specific only for cyanomyophage isolates as inferred from repeatedly negative PCR results against the sipho- and podo-cyanophage, as well as the non-cyanomyophages we examined (Table 1).

### Phylogenetic relationships of g20 sequences

**Fig. 1:**
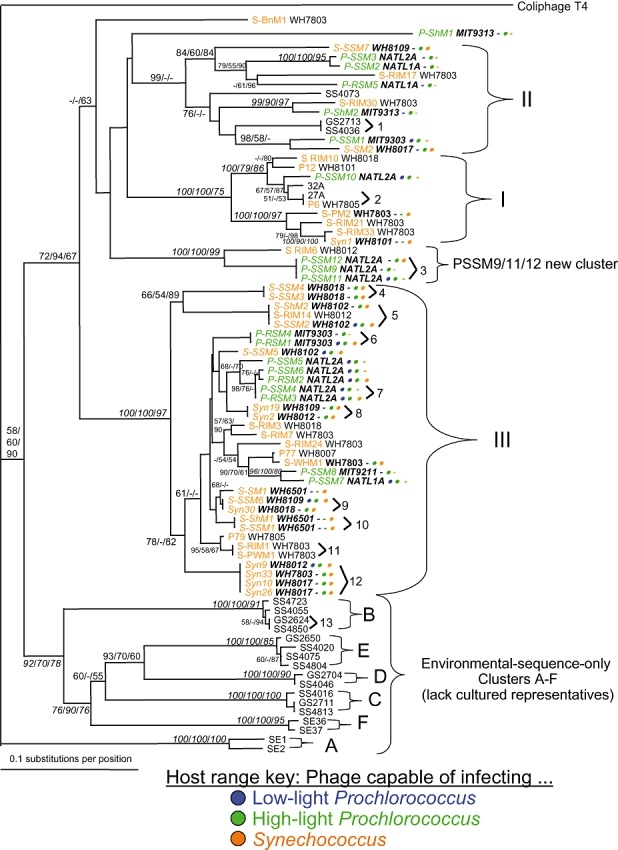
Evolutionary relationships determined using 183 amino acids of the portal protein gene (g20) amplified from cultured phage isolates (names begin with ‘S-’ or ‘P-’ and are coloured orange or green for *Synechococcus* or *Prochlorococcus* phages respectively) from this study (italicized), as well as previous studies (non-italicized), and environmental g20 sequences (names in black) (Zhong *et al*., 2002; Marston and Sallee, 2003). Clusters defined by Zhong and colleagues (2002) are as follows: clusters I–III contain g20 sequences from cultured phage isolates, while clusters A–F represent only environmental g20 sequences. Clusters containing identical g20 protein sequences are numbered with alphanumeric numbers (1–13). For cultured phages, the phage isolate names are followed by black lettering that indicates the original host strain used for isolation, while the phage host range is indicated as high light-adapted *Prochlorococcus* (green circle or dash), low light-adapted *Prochlorococcus* (blue circle or dash) or *Synechococcus* (orange circle or dash)*.* The circles represent cross-infection was observed within this group of hosts tested, whereas a dash indicates that no cross-infection was observed. Isolates not available for host range testing have no indication of their host range. The tree shown was inferred by neighbour-joining as described in the *Experimental procedures*. Support values shown at the nodes are neighbour-joining bootstrap/maximum parsimony bootstrap/maximum likelihood quartet puzzling support (only values > 50 are shown). Well-supported nodes (as defined in *Experimental procedures*) are designated by italicized support values, including six nodes that represent subclusters within the culture-containing clusters I–III. The g20 sequence from the non-cyanomyophage isolate T4 was used as an outgroup to root this tree.

We next analysed how these new g20 sequences from cultured isolates compared with selected sequences (see *Experimental procedures*) from the databases (Fig. 1). Randomly paired g20 sequence identities from this data set ranged from 59% to 100% amino acid identity, notably with some identical g20 protein sequences observed multiple times (alphanumeric clusters #1–13 in Fig. 1). This is not unprecedented: even at the level of the gene, identical viral sequences have been previously reported from vastly different aquatic environments using two separate gene markers including g20 (Zhong *et al*., 2002; Marston and Sallee, 2003; Short and Suttle, 2005) and DNA polymerase (Breitbart *et al*., 2004b; Breitbart and Rohwer, 2005).

In phylogenetic analyses, 40 of 45 g20 sequences from cyanomyophages (38 new, 7 previously published) grouped within the clusters that contain cultured representatives (I, II and III), four fell into a new monophyletic cluster (indicated by ‘PSSM9/11/12 new cluster’ on Fig. 1), and one (P-ShM1) fell onto a long branch. None fell into the previously defined (by Zhong *et al*., 2002) environmental-sequence-only clusters A–F, which were thought to be from marine cyanomyophages because of the use of isolate-designed and -tested ‘cyanophage-specific primers’. Thus either our phage culture collection is still not diverse enough to represent the g20 diversity of phages that infect marine cyanobacteria, or the sequences in the environmental-sequence-only clusters A–F represent myophages that infect other hosts. Observations made by Short and Suttle (2005) lend support to the latter. They found three g20 sequences in waters 3246 m deep in the Arctic Chukchi Sea, waters unlikely to contain cyanobacteria and their phages, which grouped with cluster A.

Given our extensive host range information for these cyanobacteria phage–host systems, we examined g20 clustering patterns for relationships with respect to the host strains upon which the phage were isolated or could cross-infect. None of the three culture-containing clusters (I, II, III) were comprised solely of g20 sequences from phages with similar hosts (Fig. 1), and no clear-cut patterns emerged when subclusters within these clusters were evaluated. This is consistent with the observations of Stoddard and colleagues (2007), who recently reported that g20 sequences could not predict the pattern of cross-resistance observed when selecting for cyanophage resistance in *Synechococcus.* Conversely, they also found that *Synechococcus* DNA-dependent RNA polymerase genotypes were not related to phage sensitivities (Stoddard *et al*., 2007). Thus for the *Prochlorococcus*/*Synechococcus/*myophage system in Fig. 1, it appears that commonly used phage and host genetic markers lack the ability to predict either the range of hosts that a phage can infect, or the range of phages to which a host is susceptible.

**Fig. 2:**
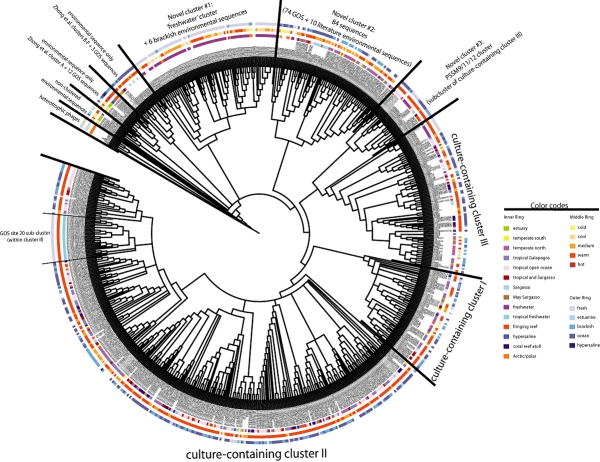
Evolutionary relationships determined using 554 base pairs of the portal protein gene (g20) from 769 available g20 sequences. Clusters defined by Zhong and colleagues (2002) are identified as culture-based clusters I–III and environmental-sequence-only clusters A–F. New clusters defined since Zhong and colleagues (2002) are indicated with the preface ‘new cluster’, a number and a brief description. The tree shown is the consensus (majority rules) tree from 11 GARLI iterations inferred using the maximum likelihood criterion (see *Experimental procedures*), with the Aeromonas phage Aeh1 g20 sequence used as an outgroup to root the tree. Three colour rings reflect the habitat type from which the g20 sequence originated. For most of these sequences (GOS sequences), there is ribotype dot-blot and metagenomic information about the microbial community structure at the site, while for non-GOS sequences such information was assumed where reasonable to do so (see Table 3 legend). The inner ring is the microbial community structure information listed as Rusch and colleagues (2007)-defined environmental categories, while the other two rings reflect the temperature and salinity of the original sampling site.

We next added more recently published g20 sequences to this analysis, including those from the non-PCR-based GOS metagenomics database (Rusch *et al*., 2007) and all published PCR-based environmental sequences (Fig. 2, Table 2). Only sequences of sufficient length for phylogenetic analysis were used. The majority (464 of 769) of these environmental sequences, including 401 GOS sequences, grouped in culture-containing clusters I, II and III. First we found that 13 of the 38 GOS sample sites included in our analysis lack *Prochlorococcus* and *Synechococcus* (as determined by dot-blots in Rusch *et al*., 2007), yet 75 g20 sequences from these sites fell into clusters I, II and III (Fig. 2), thought, from earlier studies, to represent myophages that infect marine pico-cyanobacteria. Thus it appears that clusters I, II and III likely represent phages that infect a diversity of hosts and are not limited to pico-cyanobacteria-dominated environments. Second, these analyses revealed that cluster II contains ∼10-fold more GOS sequences than clusters I and III (336 versus 32 and 33 respectively). If we ignore possible cloning bias, this suggests that cluster II sequences are by far the most abundant type in theenvironments sampled. Third, we note that a relatively tiny number of the GOS sequences fell into the environmental-sequence-only clusters – clusters A–F in Fig. 1– that were defined by Zhong and colleagues (2002) (Fig. 2). The 12 that fell into cluster A originated from seven sites with different physicochemical characteristics (see colour rings, Fig. 2). Even fewer sequences fell into environmental-sequence-only clusters B–F, suggesting that these types of g20 sequences are either extremely rare in the environments sampled to date, or are sequencing artefacts.

**Table 2:** Origins of the g20 sequences used in ‘meta’ phylogenetic analyses shown in Fig. 2.

# Sequences	Description	PCR-based?	Sequence label in Fig. 2	Refs
512	Environmental sequences from 42 oceanic sample sites from the GOS	N	JC#	1
56	Environmental sequences from 19 globally distributed freshwater and marine sites	Y	AY705#	2
25	Environmental sequences from Rhode Island coastal waters, USA	Y	AY259#	3
43	Environmental sequences from Lake Erie, USA	Y	DQ318#	4
47	Environmental sequences from Lake Bourget, France	Y	AY426#	5
27	Environmental sequences and mixed lysates from coastal north-western Atlantic Ocean	Y	Variable	6
51	Cultured marine cyanomyophages of variable coastal and open ocean origins	N/A	Variable	3, 7
8	Cultured non-cyanomyophages from sewage	N/A	Variable	8

The ‘PCR-based’ column indicates whether the environmental sequence was obtained by PCR or metagenomic approaches (N/A indicates that this is not applicable for sequences from cultured phage isolates). Reference code: 1, Rusch and colleagues (2007); 2, Short and Suttle (2005); 3, Marston and Salee (2003); 4, Wilhelm and colleagues (2006); 5, Dorigo and colleagues (2004); 6, Zhong and colleagues (2002); 7, this study; 8, T4-like phage genomes website http://phage.bioc.tulane.edu/

**Table 3:**
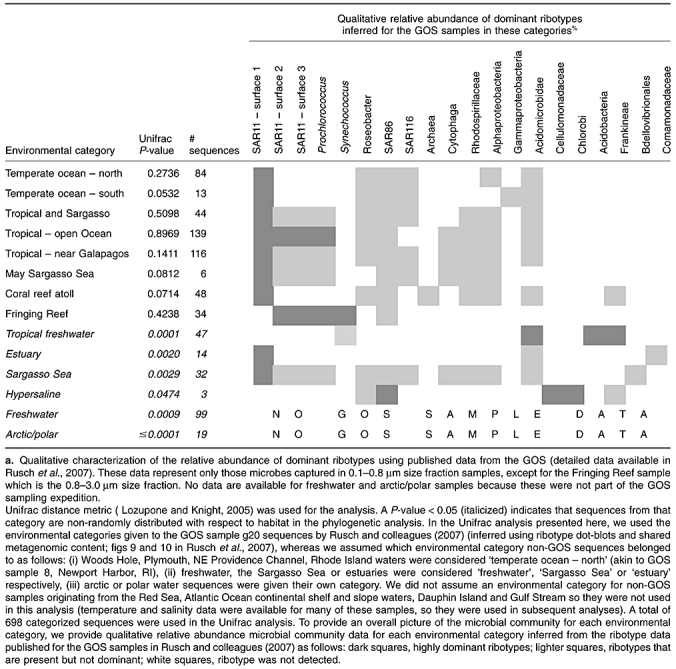
Relationship between g20 sequence clusters and the microbial community types of the original habitats from which they were collected.

This expanded data set lends support for three additional g20 lineages (Fig. 2). These include 93 sequences that group with the previously identified ‘freshwater’ cluster (Dorigo *et al*., 2004; Short and Suttle, 2005; Wilhelm *et al*., 2006; labelled as ‘new cluster #1’ in Fig. 2), 25 sequences that group with the new culture-containing P-SSM9/11/12 cluster (named after the original phage isolates forming this cluster in Fig. 1, labelled as ‘new cluster #2’ in Fig. 2) and 84 environmental sequences (74 GOS + 10 non-GOS environmental sequences, labelled as ‘new cluster #3’ in Fig. 2) of mixed biogeographic and habitat origin that form a new environmental-sequence-only cluster.

### Relationship between g20 clusters and habitat

Using Unifrac distance metric statistical tools (Lozupone *et al*., 2006), we examined the meta-g20 data set for correlates between sequence clustering and habitat descriptors, such as the microbial community type, temperature and salinity of the original sample. As a first approximation of the microbial community type, we used previously defined environmental categories originally inferred from ribotype dot-blots and metagenomic sequence data (figs 9 and 10 in Rusch *et al*., 2007) for the GOS g20 sequences, then assigned such categories where reasonable assumptions could be made for non-GOS sequences (details in Table 3 legend). We found that the g20 sequence clusters were non-randomly distributed with respect to sequences that originated from freshwater, tropical freshwater, arctic/polar, estuarine, Sargasso and hypersaline environments, while eight other environments lacked statistically significant clustering (Table 3). Beyond habitat-related properties, we also observed non-random g20 sequence distributions relative to abiotic factors, such as salinity (four of five categories significant, Table 4) and temperature (three of five categories significant, Table 5). In both cases, the outermost categories (e.g. ‘cold’ and ‘hot’, but not ‘medium’ for temperature) were significantly structured, but median categories were not. Qualitatively, some of these clustering patterns are also evident in the colour-coded rings in Fig. 2.

**Table 4:** Probability that g20 sequence clusters are non-random with respect to the salinity at the site from which they were collected.

Environmental category	Salinity (ppt)	# Sequences	Unifrac *P-*value
*Sewage*	*N/A*	*6*	*≤ 0.0001*
*Fresh*	*< 0.50*	*149*	*≤ 0.0001*
*Estuarine*	*0.5–17.99*	*6*	*0.0096*
Brackish	18–32.99	183	0.1456
*Ocean*	*33–38*	*286*	*0.0006*
*Hypersaline*	*> 38*	*8*	*0.0474*

The Unifrac distance metric (Lozupone *et al*., 2006) was used for the analysis. Salinity values, when not available from the published work, were obtained from the communicating author of the paper in which the g20 sequence was first reported. All freshwater samples were assumed to have a salinity of < 0.50 ppt. All but the sequences from brackish waters clustered non-randomly (*P*< 0.05) with respect to the habitat type as defined by salinity.

**Table 5:** Probability that g20 sequence clusters are non-random with respect to the temperature at the site from which they were collected.

Environment	Temperature (°C)	# Sequences	Unifrac *P*-value
*Sewage*	*N/A*	*6*	*≤ 0.0001*
*Cold*	*< 4.99*	*20*	*≤ 0.0001*
*Cool*	*5–14.99*	*57*	*0.2209*
Medium	15–21.99	141	0.2296
*Warm*	*22–29.99*	*467*	*0.0003*
*Hot*	*> 30*	*3*	*0.0394*

The Unifrac distance metric (Lozupone *et al*., 2006) was used for the analysis. Temperature values, when not available from the published work, were obtained from the communicating author of the paper in which the g20 sequence was first reported. All but the sequences from moderate temperatures clustered non-randomly (*P*< 0.05) with respect to the habitat type as defined by temperature.

Notably, however, clustered sequences, when significantly correlated with a habitat characteristic, always contained exceptions. For example, the ‘freshwater’ category was one of the most significantly non-random sequence categories (Fig. 2, Tables 3–5). In spite of this, the ‘freshwater’ cluster also contained 6 sequences from brackish waters, while 68 additional freshwater sequences were distributed elsewhere in the tree (light blue in the outer circle in Fig. 2). Similarly, while sequences in the ‘tropical freshwater’ category were found to be non-randomly distributed (Table 3), this is likely driven by the 24 sequences that form a well-defined subcluster within cluster II (GOS site 20 subcluster in Fig. 2). However, another 18 sequences from this same sample are scattered throughout the rest of the tree (11 in cluster II, 4 in cluster I and 3 in other clusters).

In other words, while some patterns emerge, exceptions are so frequent that one must conclude that the g20 sequence is not a good predictor of the habitat from which the phage originated. This is perhaps not surprising given the sheer abundance of phages on the planet (10^31^ phages) and the apparent promiscuity of viral–host interactions allow a lot of ‘rule breakers’ to persist. For example, not only can viral particles survive the physical challenges of extreme environmental shifts (Breitbart *et al*., 2004c), but viruses from one environment (e.g. freshwater Great Lakes) are also readily capable of infecting hosts from another environment (e.g. oceanic *Synechococcus*; (Wilhelm *et al*., 2006). Further, in coliphage T4, the g20 gene encodes a portal protein (Marusich and Mesyanzhinov, 1989) involved in functions quite removed from the direct interaction between phage and host. In contrast, the distal tail fibre gene is known to be the direct determinant of host range in T-even coliphages (Henning and Hashemolhosseini, 1994; Tetart *et al*., 1998). Thus, g20 sequence patterns might no longer correlate to host range at the fine scales (e.g. cyanobacteria and their phages) where host range ‘jumps’ could more commonly occur (e.g. by simple tail-fibre-switching *sensu*Tetart *et al*., 1998) that would de-couple host properties from vertically evolved g20 sequence lineages.

### Concluding remarks

Taken together, these data reveal that oceanic phage g20 sequence clustering patterns are, at a fine level (e.g. cyanobacteria-cyanophages), largely uncorrelated to host factors. As one zooms out to more generally consider the relationship between g20 sequences from the wild and the habitat characteristics from which they were collected, we find that they are non-randomly distributed, reflecting in some cases a connection between habitat properties, microbial community structure and phage community composition as defined by the g20 gene. We posit that the latter patterns, when evident, reflect host range-limited vertical evolution of g20 sequences, while the former reflects highly specific ‘tip-of-the-tree’ phage–host interactions that are evolutionarily disconnected from that of the g20 protein product.

## Experimental procedures

### Phage isolates

Forty-five cyanomyophages were isolated (Table 1) as described previously (Waterbury and Valois, 1993; Wilson *et al*., 1993; Marston and Sallee, 2003; Sullivan *et al*., 2003). S-PM2 and S-WHM1 were provided by W. Wilson and all S-RIM phages were provided by M. Marston. The specificity of cyanomyophage g20 primers was tested using five marine *Pseudoalteromonas* spp. bacteriophages (HER320, HER321, HER322, HER327, HER328; Wichels *et al*., 1998) that were purchased from the Felix d'Herelle Reference Center for Bacterial Viruses (contact H. Ackermann) as well as seven heterotrophic bacteriophages (IH6-φ1, IH6-φ7, IH11-φ2, IH11-φ5, CB8-φ2, CB8-φ6, CB-φ8; Zhong *et al*., 2002) kindly provided by F. Chen.

### Primer redesign

To obtain g20 PCR amplicons from myophage that would not amplify using published primers, we added degeneracies to both CPS1 and CPS8, and shifted the CPS8 primer based upon genomic sequence data from two *Prochlorococcus* myophage isolates, P-SSM2 and P-SSM4 (Sullivan *et al*., 2005), to design CPS1.1 5′-GTAGWATWTTYTAYATTGAYGTWGG-3′ and CPS8.1 5′-ARTAYTTDCCDAYRWAWGGWTC-3′.

### PCR amplification and sequencing

Previous g20 PCR primer sets [non-degenerate CPS4GC/CPS5 (Wilson *et al*., 1999) and degenerate CPS1/CPS8 (Fuller *et al*., 1998; Zhong *et al*., 2002] were designed to amplify ∼200 bp and ∼592 bp fragments, respectively, of the T4 g20 homologue in myophages.

The PCR reactions for CPS4GC/CPS5 and CPS1/CPS8 were conducted as described previously (Wilson *et al*., 1999; Zhong *et al*., 2002). Briefly, 2 μl of cyanophage lysate was added as DNA template to a PCR reaction mixture (total volume 50 μl) containing the following: 20 pmol each of a forward and reverse primers, 1× PCR buffer (50 mM Tris-HCl, 100 mM NaCl, 1.5 mM MgCl_2_), 250 μM of each dNTP and 0.75 U of Expand high-fidelity DNA polymerase (Roche, Indianapolis, IN). The PCR amplification was carried out with a PTC-100 DNA Engine Thermocycler (MJ Research, San Francisco, CA). Optimized thermal cycling conditions varied slightly from those reported as follows: CPS4GC/CPS5 required an initial denaturation step of 94°C for 3 min, followed by 35 cycles of denaturation at 94°C for 1 min, annealing at 50°C for 1 min, ramping at 0.3°C s^−1^, and elongation at 73°C for 1 min with a final elongation step at 73°C for 4 min, whereas both primer sets CPS1/CPS8 and CPS1.1/CPS8.1 required an initial denaturation step of 94°C for 3 min, followed by 35 cycles of denaturation at 94°C for 15 s, annealing at 35°C for 1 min, ramping at 0.3°C s^−1^, and elongation at 73°C for 1 min with a final elongation step at 73°C for 4 min. Systematic PCR screening using various primer sets was conducted using the same PCR reaction conditions and amplification protocol, but replacing the high-fidelity DNA polymerase with the less-expensive Taq DNA polymerase (Invitrogen, Carlsbad, CA) and only using 20 μl reactions as replicate (range 3–8) PCR reactions were pooled before sequencing to decrease PCR bias (Polz and Cavanaugh, 1998). In all cases, a 5–10 μl aliquot of PCR product was analysed in a 1.5% TAE gel stained with EtBr. The gel image was captured and analysed with an Eagle Eye II gel documentation system (Stratagene, La Jolla, CA). For purification and sequencing, replicate PCR reactions were combined, run out on a 1.5% TAE gel and purified using the QIAGEN QIAquick gel extraction kit (Qiagen, Valencia, CA). The purified PCR products were sequenced directly on both strands using the degenerate PCR primers used to obtain the product (CPS1, CPS8, CPS1.1, CPS8.1) with best results at primer concentrations ∼10-fold those suggested by the sequencing facility (40 pmol per reaction). To have greater confidence in negative PCR results, templates that did not produce amplified product were tested against optimized primer sets multiple times (data not shown). To confirm that our correctly sized amplicons from ‘positive’ PCR reactions were in fact g20 sequences, we sequenced the products. In all cases, the amplicon sequences were from g20 homologues

Where identical g20 sequences were observed in our study, we confirmed that the match was real and not the result of PCR contamination by re-amplifying and sequencing directly from fresh phage isolates (e.g. for P-SSM4, P-RSM3, S-SSM2 and ‘Syn’ phages Syn2, Syn9, Syn10, Syn26, Syn30, Syn33, Syn1, Syn19), many of which were obtained from stocks kept at a separate institution.

### Phylogenetic analysis

For the new sequences presented in Fig. 1 of this study, paired sequence data were aligned using ClustalW (Thompson *et al*., 1997) and corrected manually using the sequence chromatograms. Consensus sequences for each cyanophage isolate were then translated in-frame into amino acids. Published g20 sequences from PCR-amplified environmental clone libraries and phage isolates were screened by building preliminary neighbour-joining trees to select representative sequences that spanned the known g20 diversity and added to this data set. Multiple sequence alignments of translated amino acid consensus sequences were done with ClustalW using the Gonnet protein weight matrix, a gap opening penalty of 15 and gap extension penalty of 0.30 (although changing these penalties did not significantly alter the alignments). Phylogenetic reconstruction was done using paup 4.0 (Swofford, 2002) for parsimony and distance trees and Tree-Puzzle 5.0 (Schmidt *et al*., 2002) for maximum likelihood trees. Evolutionary distances for neighbour-joining trees were calculated based on mean character distances, while evolutionary distances for maximum likelihood trees were calculated using the JTT model of substitution assuming a gamma-distributed model of rate heterogeneities with 16 gamma-rate categories empirically estimated from the data. A heuristic search with 10 random addition replicates using the tree-bisection-reconnection branch swapping algorithm was used for parsimony trees. Bootstrap analysis was used to estimate node reproducibility and tree topology for neighbour-joining (1000 replicates) and parsimony (100 replicates) trees, while quartet puzzling (10 000 replicates) indicates support for the maximum likelihood tree. The g20 sequence from coliphage T4 was used as the outgroup taxon for all analyses.

Phylogenetic analyses of 183 amino acids from viral g20 sequence from 79 taxa yielded robust, similar trees using both algorithmic (neighbour-joining) and tree-searching (parsimony and maximum likelihood) methods. The translated g20 sequences contained phylogenetically informative regions (e.g. for parsimony analyses, 41 positions were constant, 25 were parsimony uninformative and 117 were parsimony informative). Differences between the parsimony, distance and maximum likelihood trees were limited to the branching order of the terminal nodes in a given cluster. To evaluate whether g20 sequence diversity correlated to the host-related properties presented in Fig. 1, we empirically defined a ‘well supported node’ as one where the average support across all three phylogenetic methods was 80% or greater.

### GOS g20 identification, filtering and phylogenetic analyses

Using the 549 bp g20 fragment from all available cultured isolates as queries (Table 1), we retrieved 553 sequence reads with similarity (bit score > 100) to this region of the g20 gene from the GOS databases (downloaded from http://camera.calit2.net/), then combined these GOS sequences with available published g20 sequences. The combined sequences were aligned using Clustal X and filtered to remove short, phylogenetically uninformative sequences, as well as sequences with poor quality at the ends. This manual curation left 769 total sequences (512 GOS sequences, details in Table 2) with 554 aligned nucleotide positions. Eleven maximum likelihood trees were generated using garli (Zwickl, 2006), starting from a neighbour-joining topology calculated in paup v4b10 (Swofford, 2002). Tree searching was terminated after 100 000 generations with no significantly better scoring topology, and a score improvement threshold for termination of 0.05. Topology mutation proportions were 0.1–0.2 nearest neighbour interchange and 0.8–0.9 limited SPR (subtree pruning-regrafting), with the maximum SPR range of 8–10 branches. From the 11 resulting trees, a majority-rule consensus tree (threshold 50% agreement) was generated in paup and is presented in Fig. 2.

Statistical analyses to evaluate whether g20 clustering patterns uncovered in the phylogenetic reconstructions were related to the habitat features of the original sample (e.g. microbial community type, temperature and salinity) were carried out using the Unifrac distance metric statistical tools available at http://bmf2.colorado.edu/unifrac/index.psp (Lozupone and Knight, 2005). The database and the tree file used for the analysis are provided in Supplementary Information (Files S1 and 2). Briefly, all g20 sequences were assigned to environmental categories using meta data for each sequence, with some assumptions made as described in Table 3 legend. Missing meta data for published g20 sequences were obtained where possible from the authors of the original work, as indicated in Tables 4 and 5. The patterns of these meta data were evaluated for ‘each environment separately’ in the context of a single neighbour-joining tree that included branch lengths (File S2) using Unifrac; all statistical results were similar using the *P*-test (also available at the Unifrac site, data not shown).

### Nucleotide sequence accession numbers

The nucleotide sequences determined in this study were submitted to GenBank and assigned accession numbers EU715778–15813.

## Supplementary material

The following supplementary material is available for this article online:

**File S1**.Salinity categories used in Unifrac analyses.

**File S2**.Treefree used in Unifrac analyses.

This material is available as part of the online article from http://www.blackwell-synergy.com
